# Supplementary material for: Study on the Characteristics of Vacuum-Bagged Fermentation of Apo Pickle and Visualization Array Analysis of the Fermentation Process
Source: Foods. 2023 Sep 26;12(19):3573. doi: 10.3390/foods12193573 (PMC10572875; doi:10.3390/foods12193573)
Supplement: Supplementary file 1 [file foods-12-03573-s001.zip › foods-2604554-supplementary.pdf]

Supplementary data

Study on the characteristics of vacuum-bagged fermentation of Apo pickles and visualization array analysis of the fermentation process

Jiawei Liu [1], Mengyao Wang [1], Ying Huang [1], Hai Sun [2] and Haiying Liu [1,3,*]

[1] School of Food Science and Technology, Jiangnan University, Wuxi,214000 China; 6210112050@stu.jiangnan.edu.cn (J.L.); 6200112076@stu.jiangnan.edu.cn(M.W.); 6220111044@stu.jiangnan.edu.cn (Y.H.)
[2] Jiang Xiao Yao Food Technology Co., Ltd, Suzhou 215000, China; sh@jxyjc.com
[3] State Key Laboratory of Food Science and Technology, Jiangnan University, Wuxi 214000, China
* Correspondence: liuhaiying@jiangnan.edu.cn; Tel./Fax: +86-510-85329076

Table S1. The thermal cycling conditions of PCR.

	Reagent	Volume（μl）	Cycle Step	Temperature	Time	Cycles
First round PCR procedure	gDNA	17.5-X	Initial Denaturation	94℃	3 min	1
	10× TransStart Buffer	2.5	Denaturation	94℃	10 sec	14-16cycles
	dNTPs (2.5mM each）	2	Annealing	57℃	90 sec	
	primer F	2.5	Extension	72℃	15 sec	
	primer R		Final Extension	72℃	5 min	1
	2.5U/μL TransStart Taq	0.5	Hold	4℃		
Second round PCR procedure	first round PCR roducts	25	Initial Denaturation	94℃	3 min	1
	10×TransStart Buffer	2.5	Denaturation	94℃	10 sec	10-12cycles
	dNTPs （2.5mM each）	2	Annealing	60℃	30 sec	
	INDEX primer N	3	Extension	72℃	15 sec	
	INDEX primer S	3	Final Extension	72℃	5 min	1
	1xcocktail	4	Hold	4℃		1
	2.5U/μL TransStart Taq	0.5				
	To ddH2O	50				
	library purification，QC	27				

Table S2 Nine kinds of gas-sensitive materials

Number	Name
1	Nile red [a]
2	cresol red [b]
3	methyl red [b]
4	methyl red [c]
5	bromophenol blue [a]
6	Methyl Red Sodium Salt [d]
7	phenol red [c]
8	Bromocresol purple [a]
9	Methyl Red Sodium Salt [c]

[a]: aldehyde derivatization; [b]: pH adjustment; [c]: sulfur derivatization; [d]: untreated.

Figure S1 Arrangement order of nine gas-sensitive materials

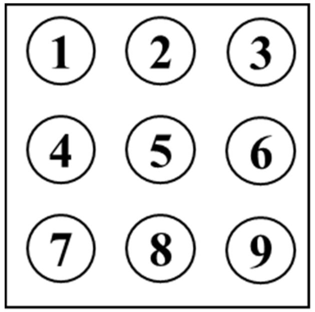

Table S3 Percentage of main volatile flavor compounds in VBA and TBA fermentation process.

No.	Compounds [a]	Formula	TBA				VBA			
			0d [b]	10d [b]	25d [b]	35d [b]	0d [b]	10d [b]	25d [b]	35d [b]
1	Acetic acid	C2H4O2	0.27±0.04	0.69±0.03	6.09±0	18.06±1.38	0.27±0.04	2.13±0.02	6.99±0.2	20.71±1.76
2	7,10,13-Hexadecatrienoic acid, (Z,Z,Z)-	C16H26O2	0.42±0.02	0.17±0.04	n.d	0.72	0.42±0.02	n.d	0.09±0.07	0.30±0.03
3	(+-)-3-Hydroxy-2-methylenepentanoic acid	C6H10O3	0.44±0.03	n.d	n.d	n.d	0.44±0.03	n.d	n.d	n.d
4	DL-Alanylglycin	C5H10N2O3	0.27±0.007	n.d	n.d	n.d	0.27±0.007	n.d	n.d	n.d
5	trans-3-Hexenoic acid	C6H10O2	0.19±0.03	n.d	n.d	0.85±0.21	0.19±0.03	n.d	0.2±0	2.71±0.2
6	Tetradecanoic acid	C14H28O2	0.06±0.03	0.01±0	0.07±0	0.52±0.06	0.06±0.03	0.20±0.02	n.d	0.6±0.05
7	Octanoic acid	C8H16O2	0.09±0.01	0.63±0.13	0.43±0.08	0.25±0.01	0.09±0.01	0.93±0.01	0.39±0.02	0.52±0.03
8	Hexanoic acid	C6H12O2	0.10±0.01	0.49±0.02	0.69±0.02	0.40±0.06	0.10±0.01	0.51±0.04	0.94±0.06	0.98±0.05
9	Nonanoic acid	C9H18O2	0.06±0.01	0.21±0.02	0.22±0.01	0.07±0.01	0.06±0.01	0.16±0.03	0.02±0.01	0.220.01
10	2-ethylexanoic acid	C8H16O2	n.d	0.19±0.06	0.48±0	0.81±0.02	n.d	n.d	n.d	n.d
11	Butanoic acid, 2-methyl-	C5H10O2	n.d	n.d	0.31±0.01	0.21±0.04	n.d	n.d	0.25±0.01	0.39±0.03
12	Pyruvic acid	C3H4O3	n.d	n.d	0.22±0.03	4.54±0.23	n.d	n.d	n.d	n.d
13	Octadecanoic acid	C18H36O2	n.d	0.02±0.01	n.d	0.49±0.02	n.d	0.01±0	n.d	0.50±0.07
14	n-Hexadecanoic acid	C16H32O2	0.02±0	0.05±0	n.d	1.17±0.80	0.02±0	0.05±0.04	n.d	0.58±0.10
15	Cyclopropanecarboxylic acid, 2,2-dimethyl-3-(2-propenyl)-	C9H14O2	n.d	n.d	n.d	n.d	n.d	n.d	n.d	0.76±0.06

16	9,12,15-Octadecatrienoic acid, (Z,Z,Z)-	C18H30O2	n.d	n.d	n.d	n.d	n.d	n.d	n.d	0.48±0.02
17	3-Methylbutanoic acid	C5H10O2	n.d	n.d	n.d	n.d	n.d	n.d	n.d	0.39±0.02
18	Benzoic acid	C7H6O2	0.11±0	n.d	n.d	0.29±0.11	0.11±0	0.04±0.01	n.d	0.21±0.08
19	1,2-Benzenedicarboxylicacid, 1-(2-butoxy-2-oxoethyl) 2-butyl ester;	C18H24O6	0.8±0.03	0.06±0	n.d	n.d	0.8±0.03	n.d	n.d	n.d
20	Methyl hexadecanoate	C17H34O2	0.75±0.02	1.35±0.09	0.29±0.02	0.49±0.07	0.75±0.02	0.97±0.04	0.3±0.03	0.66±0.07
21	Diisobutyl phthalate	C16H22O4	0.36±0.15	0.3±0	0.05±0	0.09±0	0.36±0.15	0.31±0.08	0.08±0.02	0.13±0.01
22	7,10,13-Hexadecatrienoic acid, methyl ester	C17H28O2	0.26±0.01	0.31±0.03	0.08±0	0.2±0.02	0.26±0.01	0.17±0.02	0.06±0	0.28±0.03
23	Ethyl palmitate	C18H36O2	0.22±0.02	0.49±0.04	0.27±0.01	0.51±0.05	0.22±0.02	0.58±0.02	0.47±0.05	0.76±0.08
24	3-methyl-, 3-hexenyl ester, (Z)-Butanoic acid	C11H20O2	0.88±0.08	0.52±0.08	0.58±0.11	0.35±0.06	0.88±0.08	0.21±0.03	0.41±0.1	0.21±0
25	Methyl octanoate	C9H18O2	n.d	0.65±0.13	n.d	n.d	n.d	0.44±0.04	n.d	n.d
26	Leaf acetate	C8H14O2	n.d	0.21±0.02	0.05±0	0.35±0.06	n.d	0.79±0.01	1.64±0.12	0.37±0.02
27	Phenethyl acetate	C10H12O2	0.04±0	0.05±0	0.48±0	0.37±0.05	0.04±0	0.21±0	0.76±0.04	0.59±0.03
28	Acetic acid, methyl ester	C3H6O2	n.d	n.d	1.85±0.07	n.d	n.d	0.19±0.01	n.d	n.d
29	Formic acid, octyl ester	C9H18O2	n.d	n.d	0.22±0	n.d	n.d	n.d	n.d	n.d
30	Acetic acid, hexyl ester	C8H16O2	n.d	0.03±0	0.42±0.01	0.08±0	n.d	0.08±0.01	0.36±0.09	0.07±0.02
31	Ethyl Acetate	C4H8O2	n.d	0.13±0.02	0.21±0.02	0.11±0.01	n.d	0.16±0	0.41±0.04	0.34±0.03
32	Nitric acid, pentyl ester	C5H11NO3	n.d	n.d	n.d	0.05±0	n.d	n.d	0.2±0.03	0.13±0.02
33	Isoamyl acetate	C7H14O2	n.d	0.02±0.01	n.d	n.d	n.d	0.04±0	0.43±0.25	n.d
34	Di-tert-butyl dicarbonate	C10H18O5	n.d	n.d	n.d	n.d	n.d	n.d	0.41±0.04	n.d

35	ethyl linolenate	C20H34O2	n.d	n.d	0.08±0	0.39±0.02	n.d	n.d	0.1±0.01	0.77±0.15
36	Methyl linolenate	C19H32O2	0.06±0.01	0.22±0.03	0.09±0	0.38±0.02	0.06±0.01	0.02±0.01	0.08±0	0.42±0.04
37	Methyl9,12-octadecadienoate	C19H34O2	0.05±0.01	0.09±0.01	n.d	n.d	0.05±0.01	0.08±0.01	0.03±0	0.23±0.02
38	4-Ethylphenol	C8H10O	0.34±0.22	1.28±0.04	1.33±0.23	7.44±0.42	0.34±0.22	1.73±0.01	2.83±0.22	7.61±0.19
39	o-Cresol	C7H8O	0.31±0.02	n.d	0.05±0.01	n.d	0.31±0.02	n.d	0.05±0	0.01±0
40	2-Methoxy-4-vinylphenol	C9H10O2	0.06±0.02	0.3±0.03	0.47±0.05	1.09±0.17	0.06±0.02	0.29±0.02	0.01±0	0.07±0.01
41	Phenol	C6H6O	0.03±0	0.1±0.01	0.3±0	0.26±0.02	0.03±0	0.08±0	0.28±0.02	0.27±0.02
42	Phenol, 4-methoxy-3-methyl-	C8H10O2	n.d	n.d	0.22±0.04	n.d	n.d	n.d	n.d	n.d
43	Phenol, 4-(1,1-dimethylethyl)-2-methyl-	C11H16O	n.d	n.d	n.d	n.d	n.d	n.d	0.54±0.13	n.d
44	Maltol	C6H6O3	n.d	n.d	0.01±0	0.67±0.03	n.d	n.d	n.d	0.77±0.08
45	Phenol,4-ethyl-2-methyl-	C9H12O	n.d	n.d	n.d	0.32±0.1	n.d	n.d	n.d	n.d
46	4-Ethylguaiacol	C9H12O2	0.02±0	0.01±0	0.18±0.02	0.35±0.01	0.02±0	0.02±0	0.02±0	0.16±0.02
47	2-Methoxy-5-methylphenol	C8H10O2	n.d	n.d	n.d	n.d	n.d	n.d	n.d	0.47±0.37
48	Phenol, 2,4-di-t-butyl-6-nitro-	C14H21NO3	n.d	n.d	n.d	0.01±0	n.d	n.d	0.05±0	0.22±0.02
49	3-Hexen-1-ol	C6H12O	6.72±1	4.53±0.18	5.54±0.42	4.14±0.02	6.72±1	2.43±1.92	4.04±0.59	3.36±0.53
50	Heptylalcohol	C7H16O	1.83±1.39	1.06±0.06	2.67±0.47	n.d	1.83±1.39	0.88±0.04	n.d	n.d
51	Phenethyl alcohol	C8H10O	2.2±0.18	2.67±0.09	2.75±0.02	n.d	2.2±0.18	2.69±0.02	2.75±0.22	n.d
52	1-Hexyn-3-ol	C6H10O	1.88±0.12	0.02±0.01	n.d	n.d	1.88±0.12	n.d	n.d	n.d
53	trans-2-Hexen-1-ol	C6H12O	1.64±0.39	0.04±0.03	n.d	n.d	1.64±0.39	1.65±0.15	n.d	n.d
54	1-Hexanol	C6H14O	0.83±0.15	0.81±0.13	1.27±0.03	n.d	0.83±0.15	1.09±0.01	0.81±0.01	n.d

55	1-Pentanol	C5H12O	0.62±0.02	0.52±0.02	0.81±0.11	n.d	0.62±0.02	0.6±0	0.56±0.11	n.d
56	(2Z)-2-Penten-1-ol	C5H10O	0.68±0.08	1.4±0.06	1.52±0.12	n.d	0.68±0.08	1.07±0.01	1.52±0.14	n.d
57	Ethanol	C2H6O	4.22±0.22	1.12±0.06	2.78±0.32	3.68±0.64	4.22±0.22	1.36±0.02	3.6±0.53	3.38±0.18
58	1-Penten-3-ol	C5H10O	0.45±0.19	1.5±0.1	0.69±0.01	0.1±0.01	0.45±0.19	1.16±0.02	0.82±0.02	0.19±0
59	3-Methyl-1-butanol	C5H12O	0.34±0.06	0.38±0.04	0.4±0.1	n.d	0.34±0.06	0.4±0.01	0.69±0.01	n.d
60	1-nonanol	C9H20O	0.24±0.07	0.33±0.03	0.45±0.09	n.d	0.24±0.07	0.57±0.01	0.33±0.03	n.d
61	Cyclohexanol	C6H12O	n.d	2.11±0.03	2.31±0.01	n.d	n.d	n.d	1.9±0.22	n.d
62	1-Octen-3-ol	C8H16O	n.d	1±0.09	1.45±0.05	n.d	n.d	2.09±0	1.44±0.02	n.d
63	2,7-Octadien-1-ol	C8H14O	n.d	0.73±0.11	0.43±0	n.d	n.d	1.87±0.23	n.d	n.d
64	β-Ionol	C13H22O	0.26±0.03	0.47±0.03	n.d	n.d	0.26±0.03	n.d	n.d	n.d
65	1,5-Octadien-3-ol	C8H14O	0.17±0.03	0.43±0.05	n.d	n.d	0.17±0.03	n.d	n.d	n.d
66	3,4-Dimethylcyclohexanol	C8H16O	0.06±0.01	0.48±0.01	0.06±0	n.d	0.06±0.01	0.34±0.01	0.14±0.06	n.d
67	2-Hexanol	C6H14O	n.d	1.6±0.27	n.d	n.d	n.d	n.d	n.d	n.d
68	7-Octen-4-ol	C8H16O	0.05±0.02	1.13±0.08	n.d	n.d	0.05±0.02	n.d	n.d	n.d
69	Benzenemethanol	C7H8O	0.18±0	0.96±0	0.28±0	0.01±0	0.18±0	0.17±0	0.27±0.02	n.d
70	2-Ethylhexanol	C8H18O	n.d	0.48±0.08	0.22±0.02	n.d	n.d	n.d	0.12±0.02	n.d
71	Cyclopentanol	C5H10O	n.d	n.d	1.48±0.3	n.d	n.d	0.72±0.02	n.d	n.d
72	1-Octanol	C8H18O	n.d	0.27±0.02	n.d	n.d	n.d	0.44±0.02	0.31±0.11	n.d
73	trans-2-Octen-1-ol	C8H16O	0.06±0.01	n.d	0.09±0.01	n.d	0.06±0.01	0.34±0.01	n.d	n.d
74	2-Butanol, 3-methyl-	C5H12O	n.d	0.06±0.03	1.52±0.12	n.d	n.d	n.d	n.d	n.d
75	1-Dodecanol, 3,7,11-trimethyl-	C15H32O	n.d	n.d	0.56±0.14	n.d	n.d	n.d	n.d	0.04±0.01
76	2-Undecen-4-ol	C11H22O	n.d	n.d	1.49±0.29	n.d	n.d	n.d	1.99±0.34	n.d
77	1,5-Hexadien-3-ol	C6H10O	n.d	n.d	0.23±0.02	n.d	n.d	n.d	0.2±0.05	n.d
78	2-Hexenal	C6H10O	3.9±0.64	2.26±0.22	1.7±0.15	n.d	3.9±0.64	1.75±0.05	1.67±0	0.75±0.08

79	Phenylacetaldehyde	C8H8O	1.92±0.82	0.62±0.02	0.35±0.02	0.25±0.01	1.92±0.82	0.46±0	1.29±0.03	0.43±0.05
80	trans,trans-2,4-Heptadienal	C7H10O	1.36±0.18	1.94±0.07	1.31±0.04	0.49±0.07	1.36±0.18	1.37±0.06	1.05±0.07	0.6±0.07
81	Benzaldehyde	C7H6O	0.98±0.03	2.45±0.06	0.81±0.02	1.43±0.16	0.98±0.03	1.55±0.04	1.04±0.08	1.15±0.06
82	Nonanal	C9H18O	0.92±0.05	0.42±0.05	0.96±0	0.23±0.02	0.92±0.05	0.42±0.04	0.35±0	0.23±0.05
83	Pentanal	C5H10O	0.61±0.01	0.53±0.02	0.76±0.04	0.26±0.04	0.61±0.01	n.d	0.29±0	0.23±0.01
84	Hexanal	C6H12O	0.46±0.09	0.94±0.11	0.41±0.02	0.9±0.01	0.46±0.09	0.84±0.22	0.46±0.27	0.74±0.11
85	β-Cyclocitral	C10H16O	0.56±0.05	1.29±0.05	0.44±0.01	0.28±0.03	0.56±0.05	0.86±0.07	0.48±0.05	0.37±0.02
86	Heptanal	C7H14O	0.36±0.09	0.67±0.09	0.63±0.05	0.18±0.03	0.36±0.09	0.54±0.06	0.52±0.01	0.16±0.01
87	2,6-Nonadienal,(2E,6Z)-	C9H14O	0.44±0.03	0.55±0.04	0.36±0.01	0.17±0.02	0.44±0.03	0.43±0.04	0.38±0.02	0.23±0.01
88	2-Heptenal, (E)-	C7H12O	0.16±0.05	0.61±0.1	n.d	n.d	0.16±0.05	1.08±0.01	0.5±0.08	0.15±0.01
89	2-Decenal, (Z)-	C10H18O	n.d	0.54±0.06	0.46±0.01	0.18±0.02	n.d	0.8±0.01	0.46±0.06	0.37±0.05
90	Acetaldehyde	C2H4O	n.d	n.d	n.d	n.d	n.d	2±0.27	n.d	n.d
91	2-Octenal	C8H14O	n.d	0.4±0.04	n.d	n.d	n.d	0.68±0.01	n.d	n.d
92	2-Undecenal	C11H20O	0.07±0	0.27±0.08	0.16±0	0.05±0.01	0.07±0	0.64±0.01	0.18±0	0.41±0.1
93	2-Pentenal, (E)-	C5H8O	0.17±0.04	0.31±0.01	0.7±0.06	0.34±0.04	0.17±0.04	0.22±0	0.28±0.11	0.26±0.02
94	(E)-2-Octenal	C8H14O	0.08±0.01	n.d	0.6±0.01	0.27±0.03	0.08±0.01	n.d	0.36±0.01	0.25±0.03
95	Acrolein	C3H4O	n.d	n.d	n.d	n.d	n.d	n.d	2.04±0.11	n.d
96	trans-β-Ionone	C13H20O	1.25±0.08	1.87±0.11	0.53±0.17	0.78±0	1.25±0.08	1.25±0.06	0.63±0.03	0.37±0.04
97	2-Butanone, 3-hydroxy-	C4H8O2	0.65±0.04	n.d	0.15±0	0.06±0.01	0.65±0.04	n.d	0.33±0.03	0.16±0.01
98	Geranylacetone	C13H22O	0.28±0.01	0.5±0.03	0.25±0.03	0.16±0.04	0.28±0.01	0.49±0.03	0.38±0.03	0.17±0.02
99	1-Penten-3-one	C5H8O	0.05±0.01	0.07±0	0.9±0.06	0.44±0.04	0.05±0.01	0.05±0	0.57±0.05	0.38±0.01
100	3-Pentanone	C5H10O	n.d	n.d	n.d	n.d	n.d	n.d	0.47±0.04	n.d
101	1,3-Cyclohexanedione, 2,2,5,5-tetramethyl-	C10H16O2	n.d	n.d	n.d	n.d	n.d	n.d	1.43±0.23	n.d
102	Dimethyl trisulfide	C2H6S3	2.4±0.29	n.d	0.01±0	0.02±0.01	3.95±3.7	3.82±0.02	0.03±0.01	0.03±0

103	Diacetyl sulphide	C4H6O2S	0.22±0.03	n.d	0.03±0.01	n.d	0.22±0.03	n.d	n.d	n.d
104	Methanethiol (CAS)	CH4S	0.18±0.07	n.d	2.69±0.49	n.d	0.18±0.07	n.d	n.d	n.d
105	Dimethyl sulfide	C2H6S	n.d	1.94±0.12	1.81±0.07	9.85±0.53	n.d	2.03±0.14	2.18±0.16	n.d
106	Thiocyanic acid, methyl ester	C2H3NS	0.66±0.04	0.32±0.02	0.32±0.11	0.52±0.05	0.66±0.04	n.d	0.47±0.37	0.42±0
107	Butane,2-isothiocyanato-	C5H9NS	1.87±0	0.56±0.08	0.73±0.06	0.28±0.07	1.87±0	0.16±0.08	1.99±0.36	1.28±0.1
108	2-Phenylethyl isothiocyanate	C9H9NS	4.26±0.2	0.44±0.05	0.22±0	0.32±0.02	4.26±0.2	0.19±0.02	0.6±0.01	0.64±0.03
109	1-Butene-4-isothiocyanate	C5H7NS	3.7±0.2	1.09±0.12	0.35±0.1	0.26±0.05	3.7±0.2	0.89±0.07	0.77±0.23	0.2±0.01
110	n-Heptyl isothiocyanate	C8H15NS	1.05±0.23	n.d	n.d	0.04±0.01	1.05±0.23	n.d	n.d	n.d
111	Hexane, 1-isothiocyanato-	C7H13NS	0.2±0.03	n.d	n.d	0.03±0	0.2±0.03	n.d	n.d	n.d
112	Methyl isothiocyanate	C2H3NS	n.d	0.52±0.05	n.d	n.d	n.d	0.26±0.02	n.d	n.d
113	Benzenepropanenitrile	C9H9N	2.59±0.22	1.83±0.05	1.59±0.02	2.3±0.32	2.59±0.22	1.78±0.1	1.71±0.12	n.d
114	Benzonitrile	C7H5N	0.03±0	0.07±0	0.43±0.01	0.44±0.05	0.03±0	0.02±0.01	0.52±0.07	0.73±0.23

[a] Compounds that account for more than 0.2% of all VOCs in apo pickle at a certain fermentation time.

[b] Fermentation time.

Values expressed as mean ± standard deviation for three determinations.

n.d, not detected.

Table S4 The basic OTU and species richness information.

Sample ID	PE_reads [b]	AvgLen(bp) [c]	Nochimera [d]	OTU(n)	Ace	Chao1	Shannon	Simpson
V0 [a]	90725	416.12	57135	130	146.080	143.324	3.219	0.786
V5 [a]	95129	416.08	65521	135	149.679	149.360	3.692	0.856
V10 [a]	98796	415.93	68280	139	157.840	165.840	3.492	0.848
V15 [a]	119744	416.00	94526	149	136.940	138.047	2.978	0.776
V20 [a]	202206	415.95	160886	143	137.194	137.482	2.914	0.769
V25 [a]	120750	415.94	100139	143	134.775	132.479	2.409	0.673
V30 [a]	119392	415.94	95913	124	154.960	152.631	2.708	0.720
V35 [a]	119321	415.93	101957	136	154.113	148.231	2.150	0.582
T0 [a]	90725	416.12	57135	130	146.080	143.324	3.219	0.786
T5 [a]	78530	416.17	54537	125	146.544	146.530	3.470	0.807
T10 [a]	87326	416.09	60196	126	160.369	162.550	3.859	0.890
T15 [a]	118606	416.01	96020	119	161.325	171.246	2.926	0.730
T20 [a]	128576	416.02	101819	123	159.312	162.662	3.089	0.799
T25 [a]	315623	415.98	260150	137	146.351	139.663	2.885	0.775
T30 [a]	165641	415.98	139400	121	150.973	152.032	2.854	0.733
T35 [a]	113749	416.00	97570	81	144.578	143.681	2.866	0.723

[a] V represented VBA; T represented TBA; Numbers (0, 5,10, 15, 20, 25, 30, 35) represent fermentation time. The data listed in the table are the average values of three parallel samples of the same sample.

[b] PE_reads represents the number of original PE reads.

[c] AvgLen(bp) represents the average length of the effective sequence.

[d] Nochimera represents the effective sequence after chimera removal.

Table S5 Classification results of CSA data of samples at different fermentation time by LDA.

	Fermentation Time	Backtracking validation(%)	Cross-validation(%)
Grouping by fermentation days	0d	100	100
	5d	100	91.7
	10d	100	100
	15d	100	66.7
	20d	100	100
	25d	100	100
	30d	100	91.7
	35d	100	100
	Total Accuracy	100	93.8
Grouping by fermentation stages	early fermentation	100	100
	middle fermentation	100	100
	late fermentation	100	100
	Total Accuracy	100	100
